# Discriminatory Ability and Clinical Utility of the AJCC7 and AJCC8 Staging Systems for Breast Cancer in a Middle-Income Setting

**DOI:** 10.3390/diagnostics13040674

**Published:** 2023-02-10

**Authors:** Chin-Vern Song, Carla H. van Gils, Cheng-Har Yip, Isabelle Soerjomataram, Nur Aishah Mohd Taib, Mee-Hoong See, Alexander Lim, Nur Fadhlina Abdul Satar, Nirmala Bhoo-Pathy

**Affiliations:** 1 Julius Center for Health Sciences and Primary Care, UMC Utrecht, Heidelberglaan 100, 3508 GA Utrecht, The Netherlands; 2 Ramsay Sime Darby Health Care, Jalan SS12, Subang Jaya 47500, Malaysia; 3 Cancer Surveillance Branch, International Agency for Research on Cancer, 150 Cr Albert Thomas, 69008 Lyon, France; 4 Department of Surgery, University of Malaya Medical Centre, Jalan Professor Diraja Ungku Aziz, Lembah Pantai, Kuala Lumpur 59100, Malaysia; 5 Hospital Seberang Jaya, Jalan Tun Hussein Onn, Seberang Jaya, Permatang Pauh, Pulau Pinang 13700, Malaysia; 6 Department of Clinical Oncology, University of Malaya Medical Centre, Jalan Professor Diraja Ungku Aziz, Lembah Pantai, Kuala Lumpur 59100, Malaysia; 7 Centre for Epidemiology and Evidence-Based Practice, Department of Social and Preventive Medicine, Faculty of Medicine, University of Malaya, Kuala Lumpur 50603, Malaysia

**Keywords:** breast cancer, AJCC7, AJCC8, low- and middle-income countries, prognostic performance, clinical utility

## Abstract

(1) Background: Differences in access to biomarker testing and cancer treatment in resource-limited settings may affect the clinical utility of the AJCC8 staging system compared to the anatomical AJCC7 system. (2) Methods: A total of 4151 Malaysian women who were newly diagnosed with breast cancer from 2010 to 2020 were followed-up until December 2021. All patients were staged using the AJCC7 and AJCC8 systems. Overall survival (OS) and relative survival (RS) were determined. Concordance-index was used to compare the discriminatory ability between the two systems. (3) Results: Migration from the AJCC7 to AJCC8 staging system resulted in the downstaging of 1494 (36.0%) patients and the upstaging of 289 (7.0%) patients. Approximately 5% of patients could not be staged using the AJCC8 classification. Five-year OS varied between 97% (Stage IA) and 66% (Stage IIIC) for AJCC7, and 96% (Stage IA) and 60% (Stage IIIC) for AJCC8. Concordance-indexes for predicting OS using the AJCC7 and AJCC8 models were 0.720 (0.694–0.747) and 0.745 (0.716–0.774), and for predicting RS they were 0.692 (0.658–0.728) and 0.710 (0.674–0.748), respectively. (4) Conclusions: Given the comparable discriminatory ability between the two staging systems in predicting the stage-specific survival of women with breast cancer in the current study, the continued use of the AJCC7 staging system in resource-limited settings seems pragmatic and justifiable.

## 1. Introduction

The American Joint Committee on Cancer (AJCC) staging system is a risk stratification tool used by clinicians worldwide to determine the prognosis of patients with cancer. Since its establishment, it has provided the avenue for standardized classification of cancer patients into different stages of disease, which are associated with varying risks of cancer progression and survival. The AJCC staging system has also enabled comparisons between different patient subgroups, healthcare systems and geographical regions for benchmarking cancer control efforts, including the effectiveness of screening and early detection programs.

The seventh edition of the American Joint Committee on Cancer (AJCC7) staging system for breast cancer that was published in 2010 [1] used traditional anatomical extents, comprising tumor size, number of positive lymph nodes and presence of metastasis (TNM) in determining stage. The eighth edition of the American Joint Committee on Cancer (AJCC8) staging system for breast cancer, which became effective on 1st January 2018, by far represents the most dramatic departure from previous staging classification systems [2]. Compared to the AJCC7 system, the AJCC8 staging system also requires additional information on tumor grade, as well as expression of estrogen receptor (ER), progesterone receptor (PR) and human epidermal growth factor 2 receptor (HER2). In addition, the AJCC8 system prefers the incorporation of multigene panel testing (Oncotype DX) in T1-T2, node-negative, hormone receptor positive and HER2-negative tumors. Transition from the AJCC7 to the AJCC8 classification system has therefore led to substantial changes in breast cancer staging, as now tumors can be upstaged or downstaged depending on their molecular characteristics [3].

Staging systems for cancer are continuously updated, incorporating new discoveries on clinical prognostic factors to further refine risk stratification [4]. Therefore, the newer AJCC8 classification system needs to provide a degree of benefit that can justify its added complexity, namely the need for additional data on molecular characteristics when compared to the simpler AJCC7 system. Furthermore, the AJCC8 staging system assumes that all patients have received state-of-the-art loco-regional and systemic therapies, as indicated clinically [5]. In short, with the newer AJCC8 staging system, there is now a greater emphasis on access to biomarker testing and cancer therapy.

External validation of the new AJCC8 staging system in several countries including the United States, Singapore and South Korea seem to suggest that it is more accurate than the older AJCC7 system in prognostication of women with breast cancer [6,7,8]. However, there are questions that remain. Namely, prior validation studies were largely conducted in high-income settings. The findings, therefore, may not necessarily be applicable in low- and middle-income settings (LMICs) where lack of access to biomarker testing and anti-HER2 therapy may negatively affect the prognostic accuracy of the AJCC8 staging system [9].

We therefore compared the prognostic accuracy of AJCC7 and AJCC8 staging systems in a middle-income setting with limited access to anti-HER2 therapy. The clinical utility of the AJCC8 system was also gauged by examining the number of patients who could not be staged using it.

## 2. Materials and Methods

**Figure 1:**
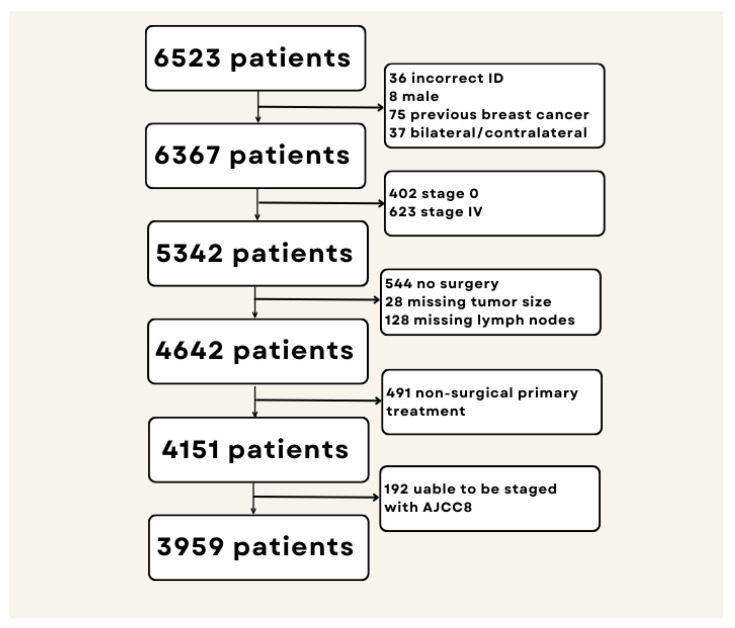
Selection flowchart of the study population.

The data for this study was obtained from two tertiary medical centers in Malaysia, Sime Darby Medical Centre (SDMC) and University Malaya Medical Centre/University Malaya Surgical Centre (UMMC/UMSC), both part of the UMMC Breast Cancer Registry, which is a prospective hospital-based registry of consecutive women with newly diagnosed breast cancer. Details of the registry have been described elsewhere [10]. Ethical approvals were obtained from the respective institutional review boards. In the current analysis, Malaysian women diagnosed with primary breast cancer from 1st January 2010 up to 31st December 2020 were included. Patients were followed-up until the end of the study, 31st December 2021, where vital status from any cause of death was obtained by linking patients’ national identity card numbers with the National Mortality Register. Women with stage 0 disease (AJCC7) and stage IV disease (AJCC7), as well as those who did not undergo surgery as primary treatment or had missing data on pathological tumor size or axillary lymph nodes status, were excluded (Figure 1).

Study variables that were extracted from the hospital-based registry included tumor size, number of positive axillary lymph nodes, tumor grade, ER status, PR status, HER2 status, sociodemographic characteristics (age at diagnosis, self-reported ethnicity and center of treatment), type of surgery (mastectomy and breast conserving therapy) and type of adjuvant therapy (chemotherapy, radiotherapy, hormone therapy and targeted therapy). ER and PR were defined as positive if more than 1% of tumor cells stained positive on immunohistochemistry, and HER2 was positive if it was graded 3+ on immunohistochemistry. If HER2 was graded 2+ on immunohistochemistry (equivocal result), silver in situ hybridization (SISH) or fluorescence in situ hybridization (FISH) was carried out to determine if HER2 overexpression was present or absent. 

All women were initially staged using the AJCC7 system [1], and subsequently restaged using the AJCC8 pathological staging system [3]. The proportions of patients whose cancer stages remained the same or changed were then estimated. A change could be an upstage, referring to a patient having a higher AJCC8 stage compared to the AJCC7 stage, or a downstage, referring to a patient having a lower AJCC8 stage compared to the initial AJCC7 stage.

Statistical analysis was done using R version 4.2.0, and survival analysis was done using the survival package. Kaplan Meier survival curves according to stage were plotted for the AJCC7 and AJCC8 classification systems. Follow-up started from the date of diagnosis of breast cancer until death from any cause (overall survival) or censoring (31st December 2021), whichever came first. Cox proportional hazards models were used to estimate the hazard of all-cause death. Separate models for AJCC7 and AJCC8 were created, and Harrell’s Concordance index was used to compare the discriminatory ability of both models. The main measure of interest was the concordance-index of the models containing only the AJCC7 or AJCC8 staging system. Subsequently, we also performed multivariable analyses that included important additional prognostic variables for breast cancer, namely age at diagnosis, ethnicity, center of treatment, type of surgery and receipt of adjuvant therapy (yes or no for chemotherapy, radiotherapy, hormone therapy and targeted therapy). 

Since data on cause of death were not available, we estimated relative survival using the relsurv package in R [11]. Relative survival is the ratio of overall survival observed in women with breast cancer in our study versus the survival that would have been expected had they been subjected only to the background mortality rates of the general population. For breast cancer, it has previously been shown that relative survival and cancer-specific survival estimates are similar [12]. Lifetables of the general Malaysian population (stratified by age, year of diagnosis, sex and ethnicity) were obtained from the National Statistics Department of Malaysia to provide the background mortality estimates. A multiplicative regression model in relative survival was applied as described by Anderson et al. [13]. 

All variables except age, tumor size and positive lymph nodes were categorical. Median values and interquartile ranges (IQR) were reported for continuous data. The proportional hazards assumption was checked using the cox.zph function (Table S1). Given the absence of an unbiased statistical test to compare two concordance-indexes [14], we compared the confidence intervals of the concordance-indexes as was done in a previous study [7]. The percentile bootstrap method was utilized to estimate the 95% confidence interval (CI) for the concordance-indexes, using the boot package in R. 

## 3. Results

A total of 4151 patients were included in this study. The median age at diagnosis was 54 years (IQR: 46–63), and most patients were of Chinese ethnicity (78.3%) (Table 1). The median tumor size at diagnosis was 22mm (IQR: 15–30) and the median number of positive lymph nodes was 0 (IQR: 0–2). Approximately two thirds of patients had had a mastectomy. The percentage of patients who were treated with chemotherapy, radiotherapy, hormone therapy and targeted therapy was 49.8%, 53.4%, 64.4% and 6.6%, respectively.

Of the 4151 patients who were staged with the AJCC7 staging system, 192 (4.6%) could not be staged using the AJCC8 staging system as they lacked data on biomarker status (Table 2). A total of 9 patients had missing data on ER expression, 10 on PR expression and 84 on HER2 expression, whereas 119 patients did not have data on tumor grade. This left 3959 patients for further analysis. 

**Figure 2:**
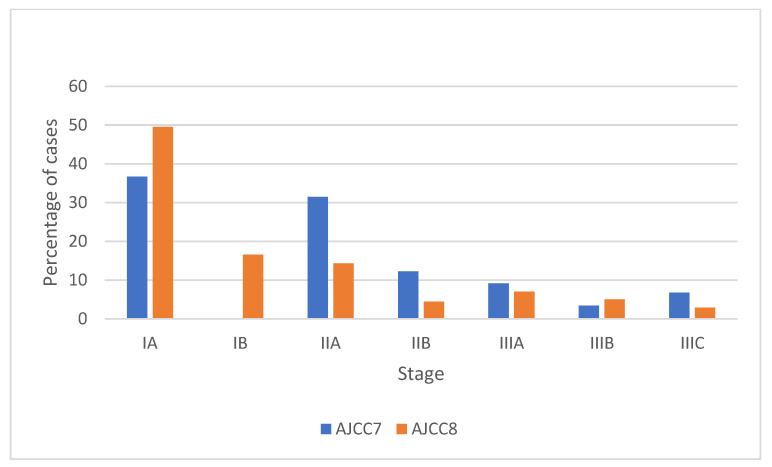
Stage distribution of AJCC7 and AJCC8 (AJCC7 in blue, AJCC8 in orange).

There was a large stage shift noted across all stages when transitioning from the AJCC7 to the AJCC8 system (Table 2, Figure 2). Overall, a larger proportion of patients were downstaged (36.0%, 1494 women) compared to the proportion of patients who were upstaged (7.0%, 289 women). The highest proportion of cancer downstaging was seen in patients who were originally classified as having stage IIIC disease under the AJCC7 system. The highest proportion of patients who were upstaged were those originally classified as having stage IIIB breast cancer. 

**Figure 3:**
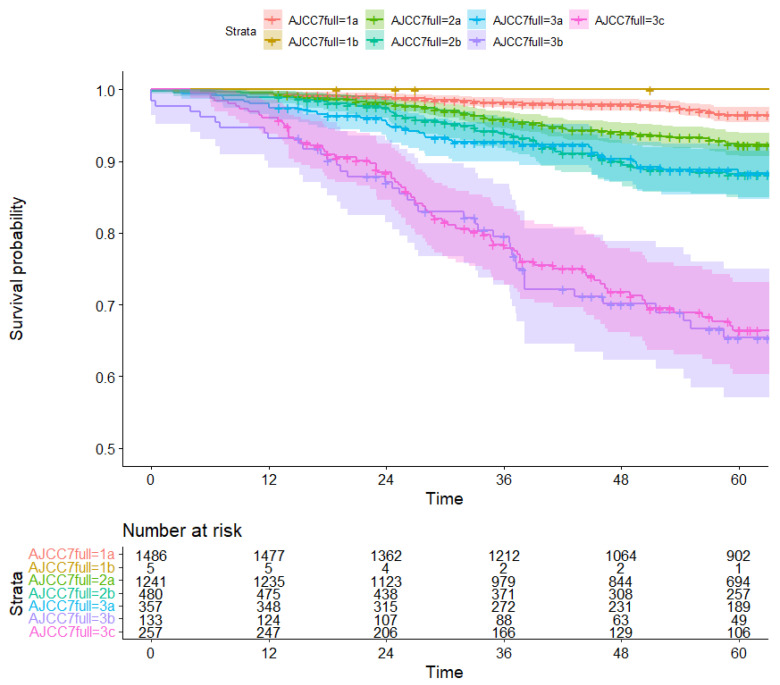
Kaplan Meier survival curve for overall survival (deaths from any cause) by AJCC7 stage (time in months).

**Figure 4:**
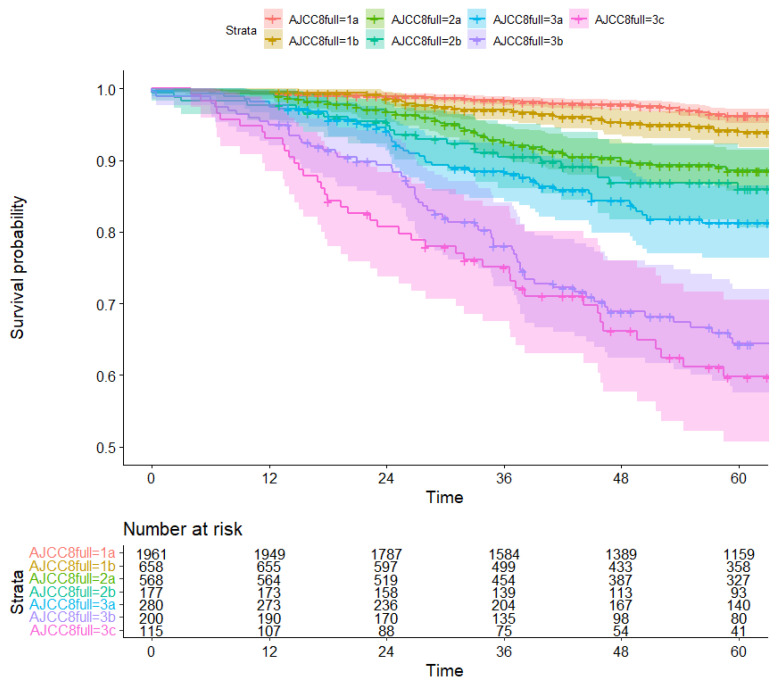
Kaplan Meier survival curve for overall survival (death from any cause) by AJCC8 stage (time in months).

Median follow-up time was 67 months (IQR: 39–100). The five-year overall survival rate varied between 97% (95% CI: 95%–98%) for stage IA and 66% (95% CI: 60%–73%) for stage IIIC for AJCC7, and 96% (95% CI: 95%–97%) for stage IA and 60% (95% CI: 51%–70%) for stage IIIC for AJCC8 (Figure 3 and Figure 4, Table S2). Women who were downstaged had a higher five-year overall survival rate than was predicted based on their original stage. For example, women who would have been classified as having stage IIIA breast cancer under the AJCC7 system were reclassified to stage IB under the new AJCC8 staging system. The corresponding five-year survival rate in the 150 patients who experienced this phenomenon was 95%, higher than the 88% that would have been expected for stage IIIA under AJCC7. Conversely, upstaged patients had a generally worse five-year overall survival than was predicted based on their original stage (Table 3, Figure S1).

Apart from stage, other prognostic factors for overall survival were age at diagnosis, center of treatment, and receipt of chemotherapy, radiotherapy, hormone therapy and targeted therapy (Table 4). The concordance-index of the model using only the AJCC7 variable was 0.720 (0.694–0.747), while the concordance-index of the AJCC8-only model was 0.745 (0.716–0.774), indicating that both staging systems on their own offered reasonably good discriminatory ability. In the multivariable models, where other variables (age, ethnicity, center of treatment, type of surgery, chemotherapy, radiotherapy, hormone therapy and targeted therapy) were also included to predict overall survival, the concordance-index of the AJCC7 model was 0.799 (0.777–0.829) while the concordance-index of the AJCC8 model was 0.795 (0.773–0.825), indicating improved prognostic performance. 

The relative survival models showed that age at diagnosis, center of treatment and receipt of chemotherapy, radiotherapy, hormone therapy and targeted therapy were significant prognostic factors (Table 5). Here, the concordance-index of the univariable model using only the AJCC7 variable was 0.692 (0.658–0.728) while the concordance-index of the AJCC8-only model was 0.710 (0.674–0.748). In multivariable analyses, the concordance-index of the AJCC7 model was 0.792 (0.769–0.825) while the concordance-index of the AJCC8 model was 0.788 (0.764–0.820).

## 4. Discussion

Our study in an upper-middle-income country has shown a significant shift in staging when migrating from the AJCC7 to the AJCC8 classification system, with a larger proportion of patients being downstaged compared to patients who were upstaged. About 5% of patients could not be staged with the AJCC8 classification system, mostly due to missing data on tumor grade or HER2 expression. This in turn implies that the AJCC8 staging system remains practical in the current study setting. Importantly, we showed that both the AJCC7 and AJCC8 staging systems offered reasonably good prediction of patients’ overall survival and relative survival. This predictive ability was further improved when other demographic and clinical variables were taken into consideration. Moreover, the 95% confidence intervals of the concordance-indexes of the AJCC7 and AJCC8 staging systems were found to largely overlap with each other. Hence, our study findings appear to suggest that both the AJCC7 and AJCC8 breast cancer staging systems offer reasonably good and similar discrimination when used in a middle-income setting.

In line with our results, a recent study conducted in Colombia, a middle-income country in South America [15], also showed that a higher proportion of patients were downstaged (40.3%) when using the AJCC8 classification system compared to the AJCC7 system. Here, the concordance-index of the AJCC7 and AJCC8 models, adjusted for age and adjuvant treatment, was 0.731 and 0.726, respectively. Findings of studies conducted in Singapore, Japan and South Korea, which are high-income Asian countries, also showed findings similar to ours [6,8,16]. On the other hand, a study using the Surveillance, Epidemiology, and End Results (SEER) database in the United States observed that the model for the AJCC8 staging system had a concordance-index of 0.814 (CI 0.807 to 0.822) compared to the corresponding model for the AJCC7 staging system, which had a concordance-index of 0.767 (CI 0.759 to 0.776) [7], with death from breast cancer as an endpoint. 

While it is difficult to compare the concordance-indexes between studies due to the variations in the variables that were included in the prognostic models, as well as in the endpoints, the differences in the prognostic performance of the models does not appear to be substantial when comparing countries with different income statuses, amid variations in access to anti-HER2 therapy. For instance, a study conducted in a high-income country (Singapore) where the demographic profiles of patients were similar to the present study also revealed findings similar to ours, although access to anti-HER2 therapy was high in Singapore [8]. Therefore, it appears that access to anti-HER2 therapy does not impede the prognostic accuracy of the AJCC8 staging system. Furthermore, even in a study where the AJCC8 system performed significantly better than the AJCC7 system [7], the magnitude of difference in the concordance-index was small, with the AJCC7 system still performing reasonably well.

In the present study, data on ER and PR expression were available for almost all patients. In our case, the biggest barrier in staging patients using the AJCC8 system stemmed from missing data on HER2 status and tumor grade. Our findings are comparable to a previous study conducted in South Africa, an LMIC, where the proportion of missing data for tumor grade and HER2 expression was greater than the proportion of missing data for ER expression and PR expression [17]. In LMICs, missing data on these biomarkers may be explained by a lack of financial resources and a shortage of pathologists [18]. Furthermore, missing data on HER2 expression may also be explained by poor access to HER2 testing as well as to trastuzumab in the LMICs [19].

To this end, implementation of the AJCC8 staging system in LMICs is expected to add a series of new challenges to the collection of data on breast cancer for cancer registries. Incomplete staging is a common challenge in many registries, particularly in resource-limited settings. For instance, around 50% to 70% of anatomical staging data for breast cancer were found to be missing in India and Zimbabwe, respectively [20]. The situation is likely to be worse if AJCC8 staging is implemented in such regions. As previously mentioned, obtaining data on tumor grade and HER2 expression status remains the biggest obstacle to the implementation of the AJCC8 staging system in LMIC settings where access to pathology services tends to be limited [9,18]. Regardless, incomplete data for anatomical variables such as tumor size and lymph node involvement remains a major challenge in these settings, and hence should be addressed as a top priority [20].

Incomplete cancer staging may lead to difficulties in national cancer surveillance programs and international benchmarking of cancer control efforts [21]. A new staging system will also have different stage-specific survival, rendering temporal or geographical comparison futile [22]. Missing biomarker information in historical data impedes the reclassification of cases which further compounds the problem. To solve these issues, one could theoretically use both the AJCC7 and AJCC8 systems at the same time. However, using two staging systems at the same time can lead to issues such as hampering comparisons of cancer stages at diagnosis between two or more populations [23]. In this case, where the AJCC7 classification system is at the very least non-inferior to the AJCC8 system in prognosticating women with breast cancer, the authors suggest prioritizing the use of purely anatomical staging systems such as AJCC7, UICC TNM or even essential TNM in resource-limited settings [20].

While it may seem pragmatic to continue using the AJCC7 system for the reasons mentioned above, there may be subgroups of patients who will benefit from using the AJCC8 system. For example, with the AJCC8 system, it is conceivable that the 150 patients in the current study who were downstaged from stage IIIA to stage IB breast cancer would at the very least be spared the distress of being diagnosed with more advanced disease [24]. Although the AJCC7 classification system may be preferable from a global cancer perspective given its practicality, we must not ignore that the AJCC8 staging system could offer tangible benefits to individual patients in routine clinical practice [25].

A strength of our study is its large sample size with many events, and the availability of a broad set of prognostic factors. Secondly, we are also confident in the validity of our mortality data as they were obtained via linkage with the national death registry using a personal unique ID. A limitation of the current study is that it was conducted in a relatively affluent setting in Malaysia, which may not be generalizable to other lower-resource settings, particularly in terms of the number of patients who could not be staged using the AJCC8 system. As such, it may be worthwhile for future studies to determine the proportion of patients who could not be staged with the newer AJCC staging systems in other lower-resource settings. Also, the lack of access to sentinel lymph node biopsy in our population made it impossible to account for its impact on the predictive capability of the AJCC8 staging system. It is also noteworthy that we were unable to integrate Oncotype DX when classifying patients using the AJCC8 system, as the test is expensive and not commonly conducted in our part of the world.

## 5. Conclusions

The AJCC7 and AJCC8 breast cancer staging systems both appear to demonstrate comparable and reasonably good discriminatory ability when used in an upper-middle-income setting. As AJCC7 is simpler and remains effective, it is felt that its continued use in resource-limited settings is pragmatic and justifiable until the health systems are ready for adoption of newer cancer staging systems. It is nonetheless felt that it is pertinent to investigate the psychosocial impact of transitioning to newer tumor classification systems (e.g., the AJCC8 system) for patients with cancer in routine clinical practice.

## Figures and Tables

**Table 1:** Baseline characteristics of the study population (*n* = 4167).

Characteristic	Value
Age, in years	
Median (interquartile range)	54 (46–63)
Center, *n* (%)	
University Malaya Medical Center (UMMC)	1304 (31.4)
University Malaya Surgical Center (UMSC)	360 (8.7)
Sime Darby Medical Center (SJMC)	2487 (59.9)
Missing	0
Ethnicity, *n* (%)	
Chinese	3249 (78.3)
Malay	509 (12.3)
Indian	379 (9.1)
Others	14 (0.3)
Missing	0
Tumor size (in cm)	
Median (interquartile range)	2.1 (1.5–3.0)
Number of positive lymph nodes	
Median (interquartile range)	0 (0–2)
Type of surgery, *n* (%)	
Mastectomy	2743 (66.1)
Breast conserving surgery	1403 (33.8)
Missing	4 (0.1)
Chemotherapy, *n* (%)	
Yes	2067 (49.8)
No	1752 (42.2)
Missing	331 (8.0)
Radiotherapy, *n* (%)	
Yes	2215 (53.4)
No	1628 (39.2)
Missing	308 (7.4)
Hormone therapy, *n* (%)	
Yes	2674 (64.4)
No	1154 (27.8)
Missing	323 (7.8)
Targeted therapy, *n* (%)	
Yes	274 (6.6)
No	3877 (93.4)
Missing	0

**Table 2:** Stage at diagnosis for women diagnosed with breast cancer in Malaysia from 2010 to 2020, using AJCC7 (rows) and AJCC8 (column) classification systems *.

AJCC7	AJCC8								
	IA	IB	IIA	IIB	IIIA	IIIB	IIIC	Missing	Total
IA	1333 (87.4)	153 (10.0)						39 (2.6)	1525
IB	4 (80.0)	1 (20.0)						0	5
IIA	592 (45.3)	150 (11.5)	499 (38.2)					66 (5.0)	1307
IIB	32 (6.3)	204 (40.0)	55 (10.8)	137 (26.9)	52 (10.2)			30 (5.9)	510
IIIA		150 (39.4)	14 (3.7)	40 (10.5)	108 (28.3)	13 (3.4)	32 (8.4)	24 (6.3)	381
IIIB					40 (28.2)	54 (38.0)	39 (27.5)	9 (6.3)	142
IIIC					80 (28.5)	133 (47.3)	44 (15.7)	24 (8.5)	281
Total	1961	658	568	177	280	200	115	192	4151

* Green indicates lower AJCC8 stage compared to AJCC7 stage, orange indicates higher AJCC8 stage compared to AJCC7 stage and grey indicates agreement between AJCC7 and AJCC8. Row percentages have been presented.

**Table 3:** Five-year overall survival rate among upstaged, downstaged and unchanged patients (AJCC7 to AJCC8).

AJCC7	AJCC8	Number of Patients	Number of Deaths	Survival Probability (95% CI)
IA	IA	1333	34	0.97 (0.87–1.00)
	IB	153	9	0.93 (0.89–0.98)
IB	IA	4	0	-
	IB	1	0	-
IIA	IA	592	25	0.95 (0.92–0.97)
	IB	150	4	0.96 (0.93–1.00)
	IIA	499	50	0.89 (0.86–0.92)
IIB	IA	32	1	0.97 (0.91–1.00)
	IB	204	12	0.92 (0.87–0.97)
	IIA	55	7	0.85 (0.75–0.95)
	IIB	137	19	0.85 (0.79–0.91)
	IIIA	52	9	0.81 (0.69–0.92)
IIIA	IB	150	7	0.95 (0.91–0.99)
	IIA	14	0	-
	IIB	40	3	0.895 (0.77–1.00)
	IIIA	108	20	0.794 (0.71–0.88)
	IIIB	13	0	-
	IIIC	32	6	0.788 (0.64–0.94)
IIIB	IIIA	40	4	0.891 (0.79–0.99)
	IIIB	54	18	0.644 (0.51–0.78)
	IIIC	39	18	0.448 (0.27–0.63)
IIIC	IIIA	80	12	0.809 (0.71–0.91)
	IIIB	133	45	0.611 (0.52–0.70)
	IIIC	44	17	0.581 (0.43–0.74)

**Table 4:** Association between stage and overall survival among patients with breast cancer by staging system (AJCC7 and AJCC8), and concordance for AJCC7 and AJCC8 staging to predict overall survival.

	AJCC7				AJCC8			
	Univariate		Multivariate		Univariate		Multivariate	
AJCC-Only Model	Hazard Ratio	*p*-Value	Hazard Ratio	*p*-Value	Hazard Ratio	*p*-Value	Hazard Ratio	*p*-Value
Stage								
IA	1		1		1		1	
IB	*		*		1.65 (1.07–2.53)	0.02	2.10 (1.31–3.36)	<0.01
IIA	2.26 (1.56–3.28)	<0.01	2.04 (1.36–3.07)	<0.01	3.33 (2.32–4.78)	<0.01	2.97 (1.92–4.60)	<0.01
IIB	3.61 (2.39–5.45)	<0.01	4.63 (2.93–7.32)	<0.01	4.23 (2.60–6.90)	<0.01	5.94 (3.40–10.38)	<0.01
IIIA	3.68 (2.36–5.73)	<0.01	4.89 (2.95–8.09)	<0.01	5.75 (3.91–8.47)	<0.01	7.83 (4.93–12.43)	<0.01
IIIB	12.62 (8.20–19.41)	<0.01	13.74 (8.39–22.51)	<0.01	12.03 (8.44–17.13)	<0.01	14.91 (9.56–23.24)	<0.01
IIIC	11.97 (8.22–17.44)	<0.01	14.93 (9.59–23.23)	<0.01	14.43 (9.70–21.48)	<0.01	14.07 (8.39–23.60)	<0.01
Age (per 1-year increase)			1.02 (1.00–1.03)	<0.01			1.01 (1.00–1.02)	0.05
Ethnicity								
Chinese			1				1	
Malay			1.29 (0.92–1.82)	0.14			1.32 (0.94–1.86)	0.11
Indian			0.82 (0.54–1.23)	0.33			0.89 (0.59–1.35)	0.59
Other			1.67 (0.23–12.03)	0.61			1.87 (0.26–13.49)	0.53
Center								
UMMC			1				1	
UMSC			0.75 (0.47–1.19)	0.22			0.69 (0.43–1.11)	0.13
SDMC			0.52 (0.38–0.69)	<0.01			0.53 (0.40–0.71)	<0.01
Type of surgery								
Mastectomy			1				1	
Breast conserving surgery			0.96 (0.68–1.35)	0.82			0.90 (0.64–1.28)	0.57
Adjuvant treatment (compared to not receiving the corresponding adjuvant treatment)								
Chemotherapy			0.59 (0.44–0.80)	<0.01			0.54 (0.40–0.73)	<0.01
Radiotherapy			0.64 (0.47–0.89)	<0.01			0.64 (0.46–0.88)	<0.01
Hormone therapy			0.34 (0.27–0.44)	<0.01			0.55 (0.41–0.72)	<0.01
Targeted therapy			0.39 (0.19–0.80)	<0.01			0.40 (0.19–0.83)	0.01
Concordance	0.720 (0.694–0.747)		0.799 (0.777–0.829)		0.745 (0.716–0.774)		0.795 (0.773–0.825)	

* too few patients in this stage.

**Table 5:** Association between stage and overall survival among patients with breast cancer by staging system (AJCC7 and AJCC8), and concordance for AJCC7 and AJCC8 staging to predict relative survival.

	AJCC7				AJCC8			
AJCC-Only Model	Hazard Ratio	*p*-Value	Hazard Ratio	*p*-Value	Hazard Ratio	*p*-Value	Hazard Ratio	*p*-Value
Stage								
IA	1		1		1		1	
IB	*		*		1.79 (1.17–2.75)	<0.01	2.09 (1.31–3.34)	<0.01
IIA	2.13 (1.47–3.09)	<0.01	2.05 (1.37–3.09)	<0.01	3.37 (2.35–4.84)	<0.01	2.94 (1.90–4.55)	<0.01
IIB	3.74 (2.47–5.66)	<0.01	4.61 (2.90–7.31)	<0.01	3.85 (2.34–6.33)	<0.01	5.64 (3.20–9.94)	<0.01
IIIA	3.64 (2.34–5.67)	<0.01	5.22 (3.15–8.63)	<0.01	5.83 (3.94–8.63)	<0.01	7.75 (4.85–12.37)	<0.01
IIIB	8.40 (5.43–13.01)	<0.01	13.47 (8.15–22.25)	<0.01	11.40 (7.99–16.27)	<0.01	14.60 (9.37–22.77)	<0.01
IIIC	13.52 (9.28–19.71)	<0.01	15.36 (9.90–23.85)	<0.01	11.97 (8.04–17.82)	<0.01	14.98 (8.92–25.15)	<0.01
Age (per 1-year increase)			0.92 (0.91–0.93)	<0.01			0.92 (0.91–0.93)	<0.01
Ethnicity								
Chinese			1				1	
Malay			1.21 (0.86–1.70)	0.27			1.25 (0.89–1.76)	0.19
Indian			0.65 (0.43–0.98)	0.04			0.71 (0.47–1.08)	0.11
Other			1.41 (1.19–10.13)	0.74			1.57 (0.22–11.33)	0.65
Center								
UMMC			1				1	
UMSC			0.62 (0.38–0.99)	0.04			0.57 (0.36–0.92)	0.02
SDMC			0.63 (0.47–0.83)	<0.01			0.64 (0.48–0.86)	<0.01
Type of surgery								
Mastectomy			1				1	
Breast-conserving surgery			1.00 (0.71–1.41)	0.99			0.93 (0.66–1.32)	0.69
Adjuvant treatment (compared to not receiving the corresponding adjuvant treatment)								
Chemotherapy			0.62 (0.45–0.84)	<0.01			0.56 (0.41–0.76)	<0.01
Radiotherapy			0.60 (0.43–0.84)	<0.01			0.60 (0.43–0.83)	<0.01
Hormone therapy			0.34 (0.27–0.44)	<0.01			0.55 (0.41–0.72)	<0.01
Targeted therapy			0.33 (0.15–0.72)	<0.01			0.35 (0.16–0.76)	<0.01
Concordance	0.692 (0.658–0.728)		0.792 (0.769–0.825)		0.710 (0.674–0.748)		0.788 (0.764–0.820)	

* too few patients in this stage.

## Data Availability

The data presented in this study are available on request from the corresponding author. The data are not publicly available as they were obtained from a hospital-based registry.

## Supplementary Materials

The following supporting information can be downloaded at https://www.mdpi.com/article/10.3390/diagnostics13040674/s1: Table S1: Assessment of the proportional hazards assumption, where a value of less than 0.05 indicates the proportional hazards assumption is violated. Table S2: 5-year overall survival rate by AJCC7 stage and AJCC8 stage. Figure S1: Kaplan Meier survival curves for 5-year overall survival rate among upstaged, downstaged and unchanged patients (AJCC7 to AJCC8).

## References

[1] Edge S.B., Compton C.C. (2010). The American Joint Committee on Cancer: The 7th edition of the AJCC cancer staging manual and the future of TNM. Ann. Surg. Oncol..

[2] Amin M.B., Edge S.B., Greene F.L., Byrd D.R., Brookland R.K., Washington M.K., Gershenwald J.E., Compton C.C., Hess K.R., Sullivan D.C. (2017). AJCC Cancer Staging Manual.

[3] Koh J., Kim M.J. (2019). Introduction of a New Staging System of Breast Cancer for Radiologists: An Emphasis on the Prognostic Stage. Korean. J. Radiol..

[4] Zanoni D.K., Patel S.G., Shah J.P. (2019). Changes in the 8th Edition of the American Joint Committee on Cancer (AJCC) Staging of Head and Neck Cancer: Rationale and Implications. Curr. Oncol. Rep..

[5] Hortobagyi G.N., Edge S.B., Giuliano A. (2018). New and Important Changes in the TNM Staging System for Breast Cancer. Am. Soc. Clin. Oncol. Educ. Book.

[6] Kim I., Choi H.J., Ryu J.M., Lee S.K., Yu J.H., Kim S.W., Nam S.J., Lee J.E. (2018). Prognostic Validation of the American Joint Committee on Cancer 8th Staging System in 24,014 Korean Patients with Breast Cancer. J. Breast Cancer.

[7] Abdel-Rahman O. (2018). Validation of the 8th AJCC prognostic staging system for breast cancer in a population-based setting. Breast Cancer Res. Treat.

[8] Wong R.X., Wong F.Y., Lim J., Lian W.X., Yap Y.S. (2018). Validation of the AJCC 8th prognostic system for breast cancer in an Asian healthcare setting. Breast.

[9] Martei Y.M., Pace L.E., Brock J.E., Shulman L.N. (2018). Breast Cancer in Low- and Middle-Income Countries: Why We Need Pathology Capability to Solve This Challenge. Clin. Lab Med..

[10] Bhoo Pathy N., Yip C.H., Taib N.A., Hartman M., Saxena N., Iau P., Bulgiba A.M., Lee S.C., Lim S.E., Wong J.E. (2011). Breast cancer in a multiethnic Asian setting: Results from the Singapore-Malaysia hopital-based breast cancer registry. Breast.

[11] Pohar M., Stare J. (2006). Relative survival analysis in R. Comput. Methods Programs Biomed..

[12] Bright C.J., Brentnall A.R., Wooldrage K., Myles J., Sasieni P., Duffy S.W. (2020). Errors in determination of net survival: Cause-specific and relative survival settings. Br. J. Cancer.

[13] Andersen P.K., Borch-Johnsen K., Deckert T., Green A., Hougaard P., Keiding N., Kreiner S. (1985). A Cox regression model for the relative mortality and its application to diabetes mellitus survival data. Biometrics.

[14] Han X., Zhang Y., Shao Y. (2017). On comparing 2 correlated C indices with censored survival data. Stat. Med..

[15] Cervera-Bonilla S., Rodríguez-Ossa P., Vallejo-Ortega M., Osorio-Ruiz A., Mendoza-Diaz S., Orozco-Ospino M., Lehmann-Mosquera C., Duarte-Torres C., Ángel-Aristizábal J., Guzmán-Abisaab L. (2021). Evaluation of the AJCC Eighth-Edition Prognostic Staging System for Breast Cancer in a Latin American Cohort. Ann. Surg. Oncol..

[16] Tang L., Ishikawa Y., Matsushita H., Jingu K. (2020). Prognostic value of the AJCC 8th edition staging system for Japanese patients treated with surgery followed by radiotherapy for breast cancer. Int. J. Clin. Oncol..

[17] Toma A., O’Neil D., Joffe M., Ayeni O., Nel C., Berg E.V.D., Nayler S., Cubasch H., Phakathi B., Buccimazza I. (2021). Quality of Histopathological Reporting in Breast Cancer: Results From Four South African Breast Units. JCO Glob. Oncol..

[18] Masood S., Vass L., Ibarra J.A., Ljung B.-M., Stalsberg H., Eniu A., Carlson R.W., Anderson B.O. (2008). Breast pathology guideline implementation in low- and middle-income countries. Cancer.

[19] Trapani D., Lengyel C.G., Habeeb B.S., Altuna S.C., Petrillo A., El Bairi K., Hussain S., Mazher S.A., Elfaham E.M., Curigliano G. (2021). The global landscape of availability, accessibility and affordability of essential diagnostics and therapeutics for the management of HER2-positive breast cancer: The ONCOLLEGE-001 survey. J. Cancer Policy.

[20] Piñeros M., Parkin D.M., Ward K., Chokunonga E., Ervik M., Farrugia H., Gospodarowicz M., O’Sullivan B., Soerjomataram I., Swaminathan R. (2019). Essential TNM: A registry tool to reduce gaps in cancer staging information. Lancet Oncol..

[21] Yang D.X., Khera R., Miccio J.A., Jairam V., Chang E., Yu J.B., Park H.S., Krumholz H.M., Aneja S. (2021). Prevalence of Missing Data in the National Cancer Database and Association With Overall Survival. JAMA Netw. Open.

[22] Tan G.H., Bhoo-Pathy N., Taib N.A., See M.H., Jamaris S., Yip C.H. (2015). The Will Rogers phenomenon in the staging of breast cancer—Does it matter?. Cancer Epidemiol..

[23] Brierley J., O’Sullivan B., Asamura H., Byrd D., Huang S.H., Lee A., Piñeros M., Mason M., Moraes F.Y., Rösler W. (2019). Global Consultation on Cancer Staging: Promoting consistent understanding and use. Nat. Rev. Clin. Oncol..

[24] Syrowatka A., Motulsky A., Kurteva S., Hanley J.A., Dixon W., Meguerditchian A.N., Tamblyn R. (2017). Predictors of distress in female breast cancer survivors: A systematic review. Breast Cancer Res. Treat.

[25] Zhu H., Doğan B.E. (2021). American Joint Committee on Cancer’s Staging System for Breast Cancer, Eighth Edition: Summary for Clinicians. Eur. J. Breast Health.

