# Supplementary material for: Discriminatory Ability and Clinical Utility of the AJCC7 and AJCC8 Staging Systems for Breast Cancer in a Middle-Income Setting
Source: Diagnostics (Basel). 2023 Feb 10;13(4):674. doi: 10.3390/diagnostics13040674 (PMC9955895; doi:10.3390/diagnostics13040674)

Supplementary materials:

Table S1: Assessment of the proportional hazards assumption, where a value of less than 0.05 indicates the proportional hazards assumption is violated

Variable	AJCC7 model	AJCC8 model
AJCC7	0.72	-
AJCC8	-	0.77
Age at diagnosis	0.26	0.22
Centre	0.15	0.14
Ethnicity	0.71	0.72
Type of surgery	0.15	0.13
Chemotherapy	0.62	0.64
Radiotherapy	0.34	0.34
Hormone therapy	0.14	0.11
Targeted therapy	0.90	0.88
Overall	0.59	0.67

Table S2: 5-year overall survival rate by AJCC7 stage and AJCC8 stage

Stage	AJCC7	AJCC8
IA	0.97 (0.95-0.98)	0.96 (0.95-0.97)
IB	-	0.94 (0.92-0.96)
IIA	0.92 (0.91-0.94)	0.89 (0.86-0.91)
IIB	0.88 (0.85-0.91)	0.86 (0.81-0.92)
IIIA	0.88 (0.85-0.92)	0.81 (0.76-0.86)
IIIB	0.65 (0.57-0.75)	0.64 (0.58-0.72)
IIIC	0.66 (0.60-0.73)	0.60 (0.51-0.70)

Figure S1: Kaplan Meier survival curves for 5-year overall survival rate among up-staged, downstaged and unchanged patients (AJCC7 to AJCC8)

a: Original stage AJCC7 IA

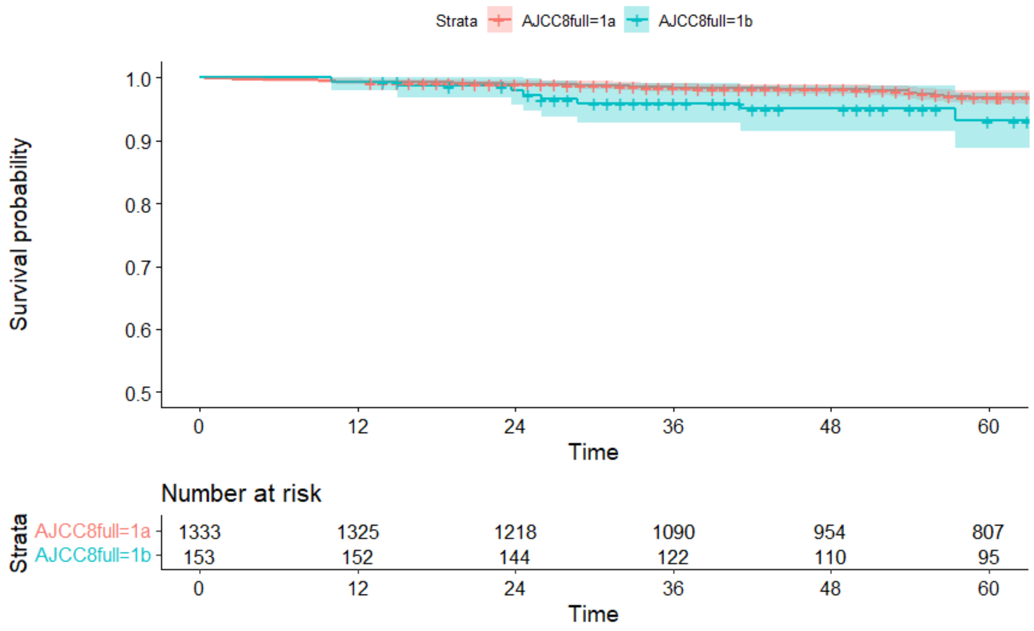

b: Original stage AJCC7 IIA

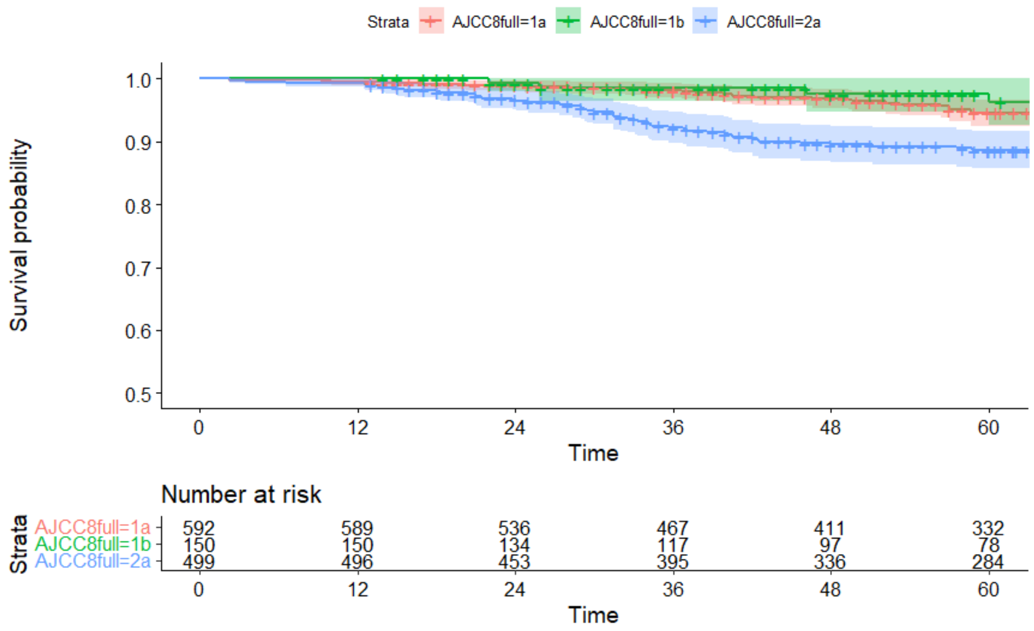

c: Original stage AJCC7 IIB

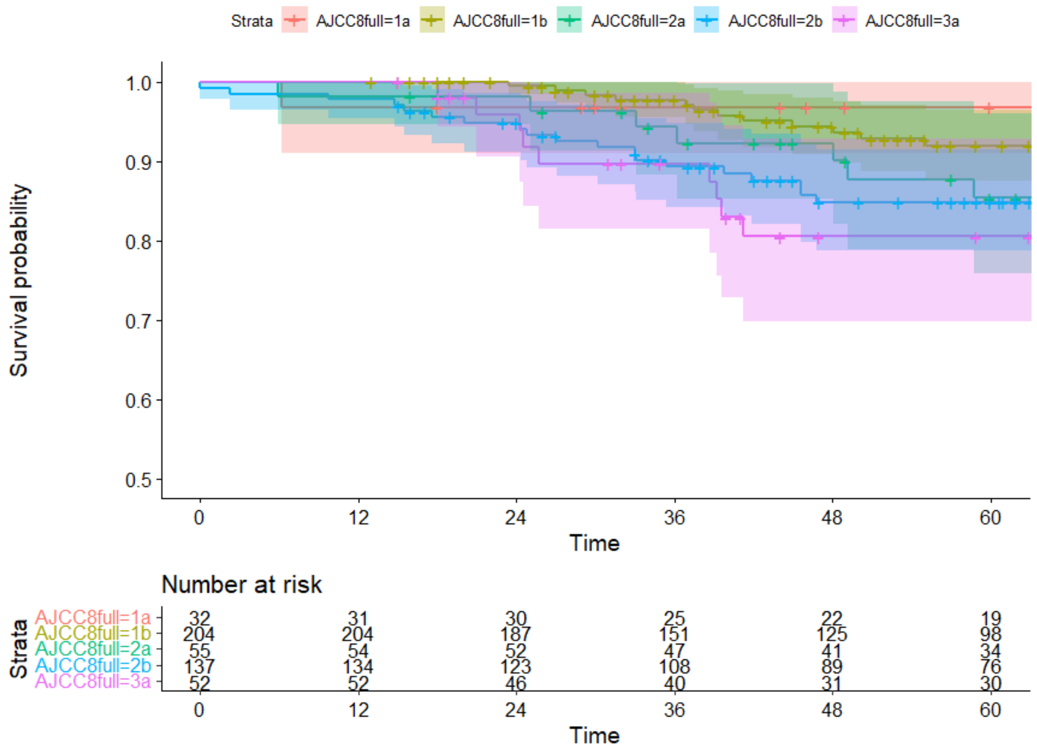

d: Original stage AJCC7 IIIA

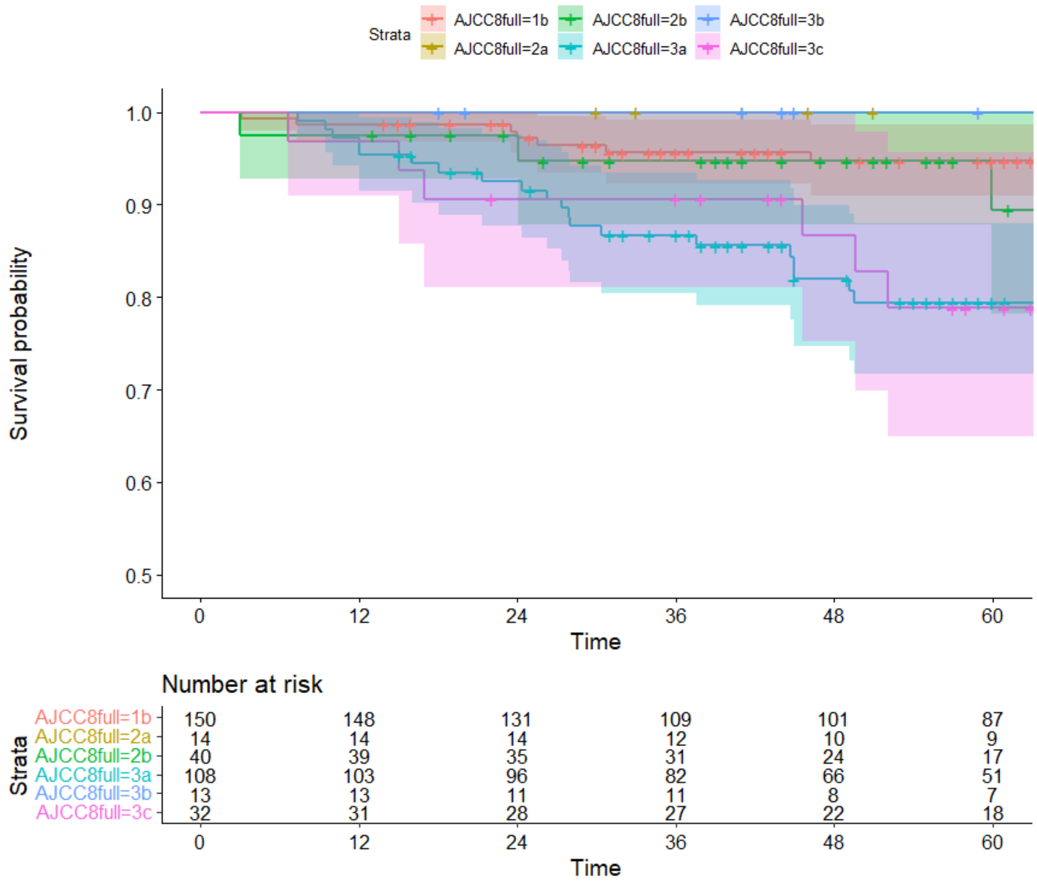

e: Original stage AJCC7 IIIB

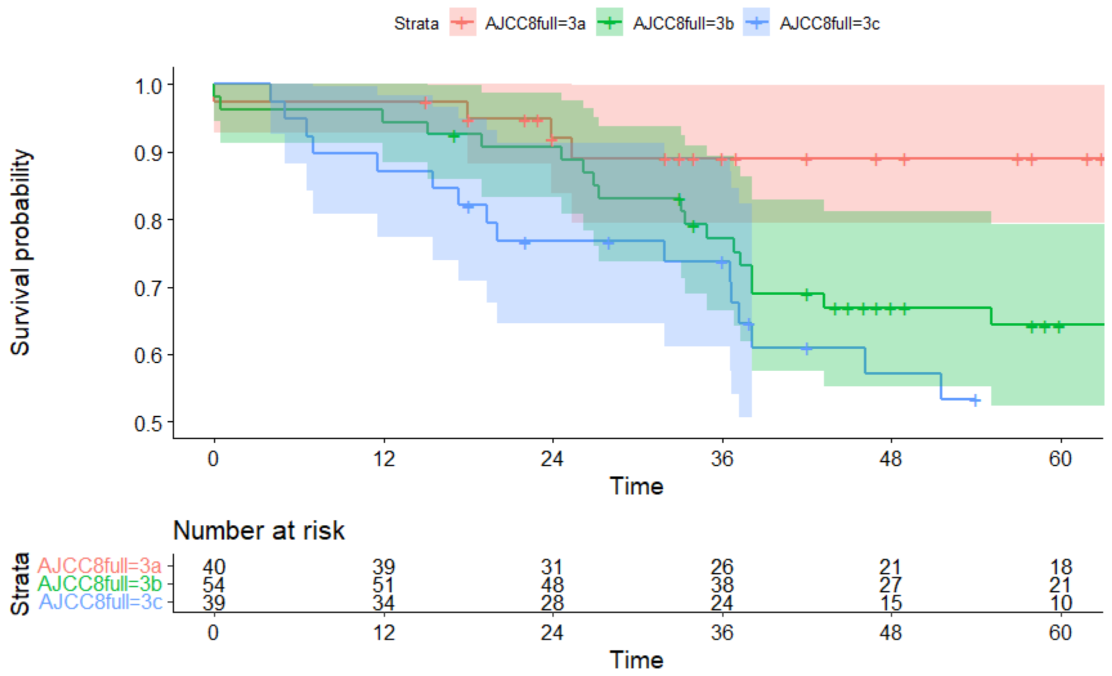

f: Original stage AJCC7 IIIC

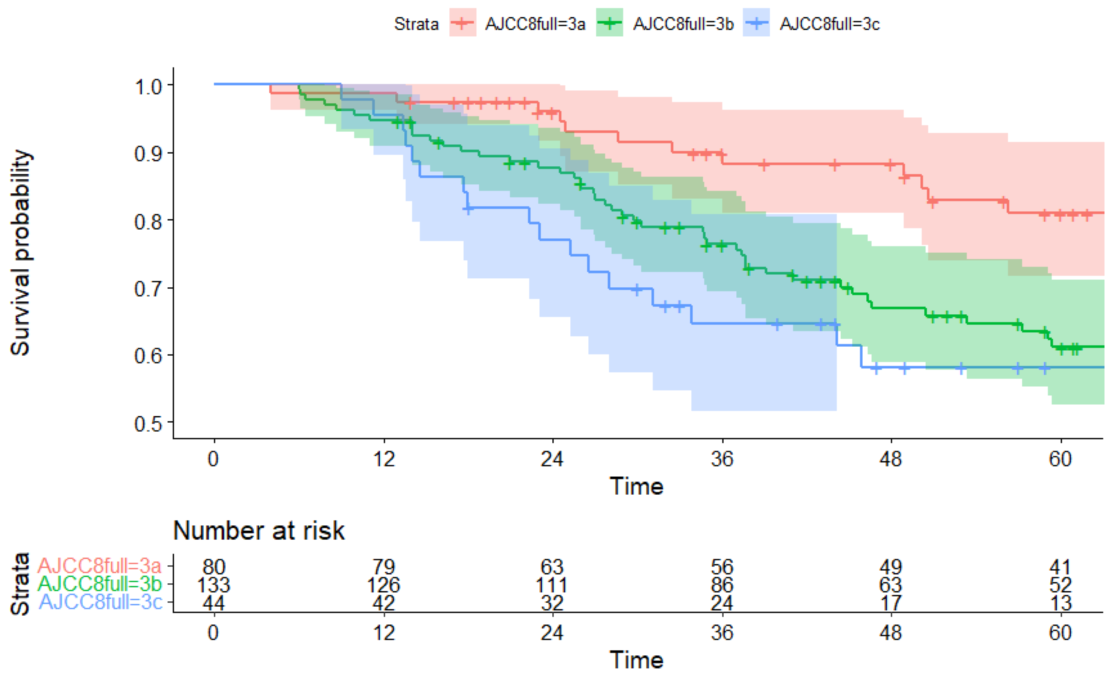

Supplement: Supplementary file 1 [file diagnostics-13-00674-s001.zip › diagnostics-2129868-supplementary.pdf]
